# Improved ECG based gating in ultra high field cardiac MRI using an independent component analysis approach

**DOI:** 10.1186/1532-429X-15-S1-W33

**Published:** 2013-01-30

**Authors:** JW Krug, G Rose, G Clifford, J Oster

**Affiliations:** 1 Chair for Healthcare Telematics and Medical Engineering, Otto-von-Guericke University of Magdeburg, Magdeburg, Germany; 2 Department of Engineering Science, University of Oxford, Oxford, UK

## Background

Cardiac gating in ultra high field (UHF) MRI is a challenging task due to the magnetohydrodynamic (MHD) effect [1]. The MHD effect is particularly severe at such field strengths and severely distorts the electrocardiogram (ECG). State-of-the-art ECG based gating methods which use the vectorcardiogram (VCG) [2] are thus prone to errors [1]. This work presents an approach which separates the ECG and the MHD signal using Independent Component Analysis (ICA).

## Methods

**Figure 1:**
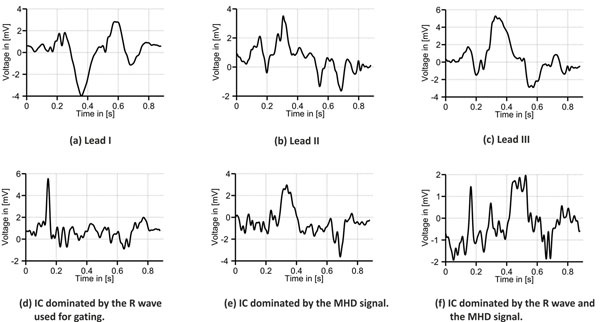
(a)-(c) ECG leads I-III recorded inside the 7T MR scanner. (d)-(e) Three ICs obtained from the 12-lead ECG. The IC shown in (d) which is dominated by the R peak is used for gating. The R peaks in (a)-(f) are aligned at 150ms.

ECGs were recorded inside a 7T MR scanner (Siemens Magnetom) using a standard 12-lead Holter ECG (Getemed, Germany). Ten data sets were acquired by measuring the ECGs from five healthy volunteers in a head first and feet first position without MR imaging. Each measurement was between 4-5min long, so a total of 2766 QRS complexes were available for analysis. Examples are given in Fig. 1(a)-(c). A FastICA algorithm was applied to the ECG signals in order to separate the ECG and MHD signals [3]. The independent component (IC) which minimizes the MHD contribution was identified using an automatic approach based on a kurtosis measurement. A QRS detection algorithm was applied to the identified IC [4]. The proposed method was compared with the VCG technique [2]. Quality was assessed in terms of sensitivity (Se) and positive prediction value (+P) of the QRS detection.

## Results

Examples for the different ICs obtained by the FastICA algorithm are depicted in Figs. 1(d)-(f). The automatic identification of the IC representing the R wave was possible in nine of the ten data sets while the 10th data set required manual identification. Regarding the QRS detection, Se and +P of 97% was achieved for all ten data sets, respectively. For the VCG based method, Se was 90% and +P reached only 48%.

## Conclusions

An ICA-based gating method has been shown to outperform the state-of-the-art VCG based gating technique for UHF MRI. As with the VCG method, the proposed ICA based method can be applied in real time, since it can be shown that the computation of the demixing matrix does not need to be updated for each new sample. Recent developments of MR safe 12-lead ECG recorders could make this method applicable in clinical practice [5].

## Funding

This work was founded by the Federal Ministry of Education (Germany, BMBF, 03IP710) and by the Royal Academy of Engineering (UK).
